# Protective responses induced by herbicide safeners in wheat

**DOI:** 10.1016/j.envexpbot.2011.12.030

**Published:** 2013-04

**Authors:** Victoria L. Taylor, Ian Cummins, Melissa Brazier-Hicks, Robert Edwards

**Affiliations:** a Centre for Bioactive Chemistry, Department of Chemistry, Durham University, Durham DH1 3LE, UK; b Centre for Novel Agricultural Products, University of York, York YO10 5DD, UK

**Keywords:** Cloquintocet mexyl, Fenchlorazole ethyl, Glutathione transferase, Glutathione peroxidase, Growth promotion, Mefenpyr diethyl, Phytoremediation

## Abstract

Safeners are agrochemicals which enhance tolerance to herbicides in cereals including wheat (*Triticum aestivum* L.) by elevating the expression of xenobiotic detoxifying enzymes, such as glutathione transferases (GSTs). When wheat plants were spray-treated with three safener chemistries, namely cloquintocet mexyl, mefenpyr diethyl and fenchlorazole ethyl, an apparently identical subset of GSTs derived from the tau, phi and lambda classes accumulated in the foliage. Treatment with the closely related mefenpyr diethyl and fenchlorazole ethyl enhanced seedling shoot growth, but this effect was not determined with the chemically unrelated cloquintocet mexyl. Focussing on cloquintocet mexyl, treatments were found to only give a transient induction of GSTs, with the period of elevation being dose dependent. Examining the role of safener metabolism in controlling these responses, it was determined that cloquintocet mexyl was rapidly hydrolysed to the respective carboxylic acid. Studies with cloquintocet showed that the acid was equally effective at inducing GSTs as the ester and appeared to be the active safener. Studies on the tissue induction of GSTs showed that whilst phi and tau class enzymes were induced in all tissues, the induction of the lambda enzymes was restricted to the meristems. To test the potential protective effects of cloquintocet mexyl in wheat on chemicals other than herbicides, seeds were pre-soaked in safeners prior to sowing on soil containing oil and a range of heavy metals. Whilst untreated seeds were unable to germinate on the contaminated soil, safener treatments resulted in seedlings briefly growing before succumbing to the pollutants. Our results show that safeners exert a range of protective and growth promoting activities in wheat that extend beyond enhancing tolerance to herbicides.

## Highlights

► Wheat treated with 3 safeners induced identical glutathione transferases (GSTs). ► Two safeners caused an enhancement in seedling growth. ► GST induction was transient, saturable and tissue dependent. ► Seeds treated with safeners were able to germinate on chemically contaminated soil.

## 1 Introduction

Safeners are an important group of agrochemicals used extensively in cereals to protect crops from damage caused by selective herbicides without compromising weed control efficacy (Davies and Caseley, 1999; Edwards et al., 2005; Hatzios, 2003; Hatzios and Burgos, 2004)^.^ The mechanism of safener action most widely accepted is that these chemicals enhance crop tolerance by inducing the expression of proteins involved in the metabolism of herbicides, thus accelerating their detoxification (Davies and Caseley, 1999; Hatzios and Burgos, 2004). Amongst these ‘safened’ enzymes, the best studied are the glutathione transferases (GSTs) which catalyze the conjugation of herbicides with the endogenous tripeptide glutathione (Cummins et al., 1997; Davies and Caseley, 1999; DeRidder and Goldsbrough, 2006). Such conjugation has been demonstrated in barley (Scalla and Roulet, 2002), maize (Fuerst et al., 1993; Scarponi et al., 2006), wheat (Cummins et al., 1997; Pascal and Scalla, 1999), the wheat progenitor *Triticum tauschii* (Riechers et al., 2003; Xu et al., 2002), and rice (Wu et al., 1999). In addition, it is known that safeners enhance the expression of other classes of detoxifying enzymes including the cytochrome P450 mixed function oxygenases (Persans et al., 2001), type 1 glucosyltransferases (UGTs) (Brazier et al., 2002) and ATP-binding cassette (ABC) transporter proteins in a range of plants (Coleman et al., 1997; Riechers et al., 2010).

**Fig. 1:**
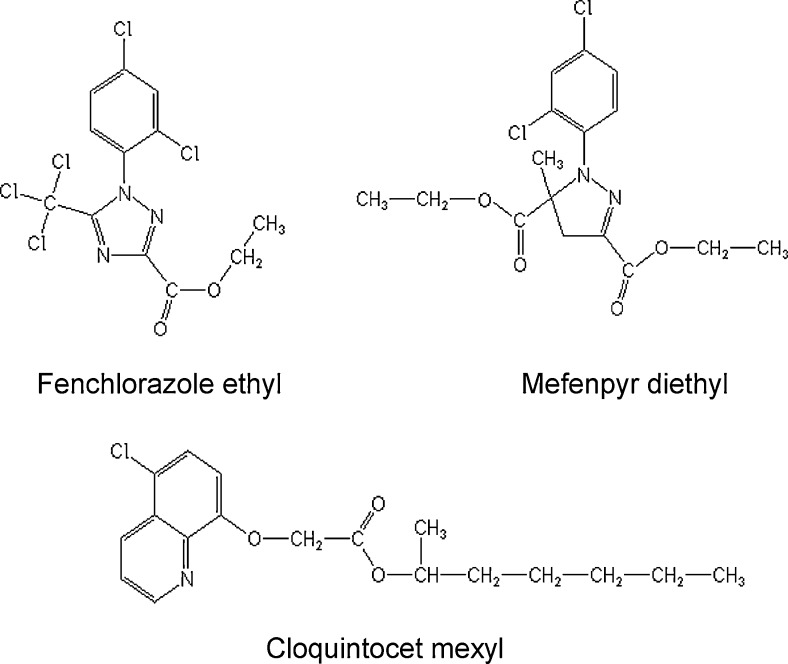
The herbicide safeners cloquintocet mexyl, fenchlorazole ethyl and mefenpyr diethyl used in wheat.

One intriguing characteristic of safeners is their apparent species specificity, with different classes of chemistry being developed for use in each of the major cereal crops (Riechers et al., 2010). In addition, for each given species a number of different safener chemistries have been developed. For example in wheat (*Triticum aestivum* L.), the quinolinoxycarboxylic acid cloquintocet mexyl, as well as the unrelated compounds mefenpyr diethyl and fenchlorazole ethyl have been widely used as post-emergence safeners (Fig. 1). All these compounds are used to safen aryloxyphenoxypropionate (AOPP) herbicides which inhibit the essential enzyme acetyl CoA carboxylase. All chemical classes of safener are known to functionally exert their protective effect by enhancing herbicide detoxification in wheat. For example, cloquintocet mexyl is known to increase the rate of hydroxylation, ether cleavage, and glucosylation of the AOPP clodinafop propargyl (Kreuz et al*.*, 1991), whilst fenchlorazole ethyl increased the cleavage by glutathionylation of the related herbicide fenoxaprop ethyl. In each case, whilst the safeners have been individually shown to enhance the activities or expression of several classes of herbicide-metabolising enzymes, there have been no reported attempts to compare the relative efficacy of these chemicals in inducing detoxification responses either qualitatively or quantitatively. It is also clear that whilst wheat safeners are predominantly used commercially to enhance tolerance towards a specific partner herbicide, this protective ability extends to multiple classes of chemistry. For example, in addition to safening the AOPP herbicide fenoxaprop ethyl in wheat, mefenpyr diethyl also increases the selectivity ratio of the unrelated sulfonyl ureas mesosulfuron-methyl and iodosulfuron methyl-sodium by enhancing their detoxification (Hacker et al., 2000).

Using cloquintocet mexyl, mefenpyr diethyl and fenchlorazole ethyl as different classes of safener (Fig. 1), we now address whether or not distinct chemistries invoke different responses in detoxifying enzymes in wheat. We then use different treatment regimes to test the longevity and dose dependence of response and evaluate the ability of safeners to protect wheat from soil borne chemical pollutants in addition to herbicides.

## 2 Materials and methods

### 2.1 Chemicals and related analysis

Safeners were purchased from Greyhound Chromatography & Allied Chemicals (Birkenhead, UK). The detergent formulant Biopower^®^ was provided by Bayer Crop Science (Cambridge, UK). To prepare cloquintocet acid, a solution of cloquintocet-mexyl (100 mg, 0.3 mmol) was dissolved in tetrahydrofuran:H_2_O (1:1, 5 mL) and treated with LiOH (30 mg, 1.2 mmol) at room temperature. The resulting solution was stirred for 3 h then diluted with water. The aqueous layer was first extracted with Et_2_O (3 × 5 mL), then acidified (pH 1) with 1 M HCl and extracted with DCM (3 × 5 mL) and finally neutralised (pH 7) with solid NaHCO_3_ and extracted with DCM (3 × 5 mL). The aqueous layer was then subjected to reverse phase (C-18) chromatography to afford the title compound as a white solid (65 mg, 93%). The sample was analysed and its authenticity confirmed by NMR using a Varian Inova instrument *δ*_H_ (500 MHz, d_6_-DMSO) 8.95 (1H, s, Ar–H), 8.57 (1H, d, *J* 8, Ar–H), 7.80 (1H, bs, Ar–H), 7.70 (1H, d, *J* 8, Ar–H), 7.20 (1H, d, *J* 8, Ar–H), 4.42 (2H, s, CH_2_) and by mass spectrometry on a Waters Acquity TQD *m*/*z* (ES^+^) 240 ([^37^Cl]M^+^) 238 ([^35^Cl]M^+^) as described previously (Brazier-Hicks et al., 2008). For the extraction of safener metabolites, wheat tissue was ground up in liquid nitrogen using a pestle and mortar, extracted in 3× (w/v) methanol and then centrifuged (3000 × *g*, 5 min) to remove cell debris. The metabolites in the resulting supernatant were analysed using an Acquity UPLC™ linked to a Waters Q-TOF Premier mass spectrometer as described (Brazier-Hicks et al., 2008).

### 2.2 Plant growth and treatment

Wheat seeds cv. Einstein were obtained from Nickerson-Advanta LTD (Lincolnshire). For post-emergence safener treatments, seeds were imbibed in water for 1 h prior to planting. For pre-emergence treatments, the seeds were soaked for 24 h in 0.1% (v/v) aqueous acetone, with or without, the safener present. For standard studies, seeds were sown on John Innes loam-based compost N° 2. For studies with chemically contaminated soil, test samples from the field were kindly donated by ConocoPhillips (Seal Sands, Middlesborough TS2 1UH). The soil samples had been monitored for metal and organic content over the 5-year period prior to assay (Table 1). The contaminated soil was then mixed with sharp sand, at a ratio of 4:1, to improve soil consistency. In each case, seeds were sown on the surface of the soil and covered with a thin layer of horticultural sand, prior to placing in an environmental chamber (Sanyo MLR-350H) at 25 °C with 60% humidity and a photoperiod of 16 h light (150 μE m^−2^ s^−1^) and 8 h dark. For post-emergence treatment, 7-day-old wheat shoots were sprayed with a hand held misting pump to run-off with 0.1% (v/v) Biopower^®^ and 0.1% (v/v) acetone containing the safener at a final concentration of 10 mg L^−1^ at an application rate of 20 mL m^−2.^ This was equivalent to the application rate of wheat safeners typically used in the field of around 15 g active ingredient Ha^−1^, albeit in a 7.5-fold greater volume of water formulant Control sprays consisted of the identical formulation without the safener. The standard post-emergence treatment protocol consisted of a single safener treatment, corresponding to field practice. Where repeated treatments were required to investigate additive effects these were applied at 24 h intervals as stated.

### 2.3 Sample preparation

All steps were carried out at 4 °C unless otherwise stated. On harvest, plant tissue was weighed, frozen in liquid nitrogen and stored at −80 °C. Frozen plant tissue was ground to a fine powder using a pestle and mortar and then extracted in 3 (v/w) 0.1 M Tris–HCl, pH 7.5, containing 2 mM ethylenediamine tetraacetic acid (EDTA), 1 mM dithiothreitol (DTT), and 5% (w/v) polyvinylpolypyrrolidone. After straining through Miracloth (Calbiochem, Nottingham, UK) followed by centrifugation (10,000 × *g*, 30 min), the supernatant was adjusted to 80% saturation with (NH_4_)_2_SO_4_ and the protein pellet recovered after re-centrifuging (10,000 × *g*, 20 min). Protein pellets were stored at −20 °C until needed, and desalted prior to use on a Sephadex spin column (4500 × *g*, 2 min), pre-equilibrated with 3 (v/w) 0.1 M Tris–HCl, pH 7.5, containing 2 mM EDTA, 1 mM DTT. A BCA™ protein assay kit (Pierce) was used for protein determination.

### 2.4 Protein analysis

GST enzyme activity was determined using by measuring the glutathione–dependent conjugation of (a) 1-chloro-2,4-dinitrobenzene (CDNB) and (b) the reduction of cumene hydroperoxide using an NADPH-linked glutathione peroxidase assay (Cummins et al., 2009). SDS-PAGE and Western blotting was carried out as using antisera raised against maize phi (GSTF1) and tau (GSTU1/2), as well as wheat lambda GSTL1 as described previously (Cummins et al., 2009).

## 3 Results

### 3.1 Comparative studies with three safeners in wheat

**Fig. 2:**
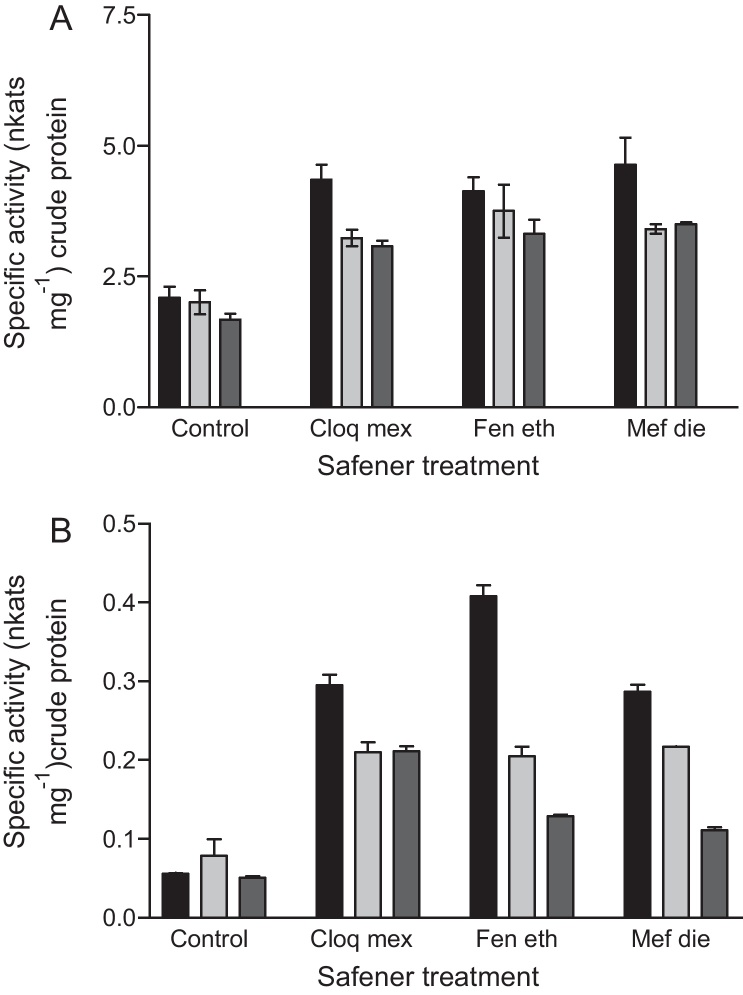
GST activity towards (A) CDNB, and (B) glutathione peroxidase activity in wheat shoots at 7 days (■), 8 days () and 9 days () after imbibing seed and subjecting shoots to daily spray treatments with the herbicide safeners, cloquintocet mexyl (Cloqmex), fenchlorazole ethyl (Feneth) or mefenpyr diethyl (Mefdie). Values represent the means of duplicate determination with the error bars showing the extent of variation between replicates.

**Fig. 3:**
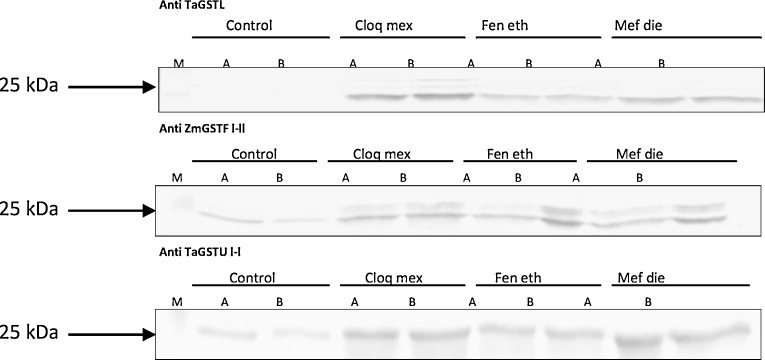
Induction of GSTs in 9-day-old wheat shoots sprayed daily with 10 mg L^−1^ solutions of the herbicide safeners, cloquintocet mexyl, fenchlorozole ethyl or mefenpyr diethyl. Protein extracts were normalised and separated by SDS-PAGE before probing with antisera raised to the lambda class GSTL (top row), phi class GST l-ll, (middle) and tau class GSTU l-l (bottom).

To study the effect of the three safeners on wheat under effectively equivalent chemically saturated conditions, seeds were imbibed and then sprayed daily with either, control formulation, cloquintocet mexyl, mefenpyr diethyl or fenchlorazole ethyl (Fig. 1). The plants were then harvested 7, 8 and 9 days after sowing and analysed for GST activities (Fig. 2) and associated polypeptide composition (Fig. 3). All three treatments caused a doubling in the enzymic conjugation of CDNB (Fig. 1A). At the later time points enzyme specific activity then declined slightly, with this effect being most pronounced with the cloquintocet mexyl and mefenpyr diethyl treatments. No significant difference (95% confidence interval) in the GST activity towards CDNB was found between the different safener treatments at any of the time points studied. The safeners had a much greater effect on glutathione peroxidase (GPOX) activity, with cloquintocet mexyl and mefenpyr diethyl inducing a six-fold increase and fenchlorazole ethyl an eight-fold increase at 7 d relative to the control (Fig. 1B). As with the GST activity towards CDNB, the GPOX activity then declined over 3 days.

From these total GST activity studies it appeared that the three safeners promoted very similar responses over the 3-day sampling period. To test the equivalence of these responses in greater detail, the plants were assayed for GST polypeptides which were recognised by antisera raised to maize phi GSTFl-ll and the wheat lambda GSTL and tau GSTUl-l (Cummins et al., 2009). These antisera were selected as all three classes were known to be induced by safeners in wheat (Cummins et al., 1997; Edwards et al., 2005; Theodoulou et al., 2003). Following SDS-PAGE and Western blotting, all three antisera recognised polypeptides of around 25 kDa (Fig. 3), which is the typical relative molecular mass of a GST subunit. The tau (GSTU) antiserum recognised polypeptides in the control samples which were enhanced to similar degrees by all three safeners. The phi anti-GSTF-serum also recognised a major polypeptide in control samples whose expression was enhanced following safener treatment. In addition, this antibody also recognised an inducible polypeptide in the safened plants which migrated with a relative molecular mass of around 27 kDa. The lambda (GSTL) antiserum identified immunoreactive polypeptides in the safened samples which were not detected in the controls, reflecting the strong link between the expression of this class of GST and chemical treatment (Theodoulou et al., 2003). Whilst minor differences in the intensity of the GSTs were determined in each case, it was concluded that the three safeners effectively promoted the same induction of the different classes in wheat.

During these trials it was noted that some of the safener treatments caused a noticeable growth promoting activity in terms of causing shoot elongation. To study this effect in greater detail, wheat seedlings were again treated daily pre- and post-emergence with the three safeners and analysed for shoot length and % dry weight content over a 14-day period (Table 2). Whilst cloquintocet mexyl did not enhance growth over this period, both mefenpyr diethyl and fenchlorazole ethyl noticeably enhanced shoot length. This was associated with a gain in the proportion of dry weight, suggesting this was due to assimilation rather than just cell expansion (Table 2).

### 3.2 GST induction studies with cloquintocet mexyl

**Fig. 4:**
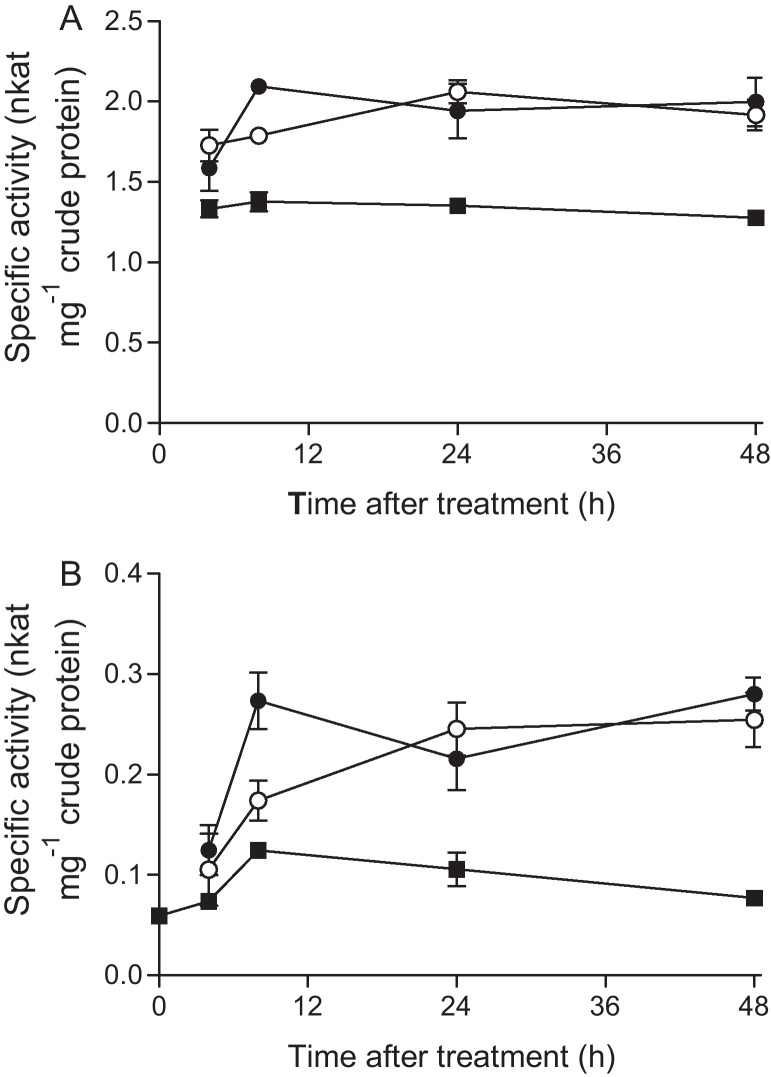
The effect on GST activity towards CDNB (A) and glutathione perioxidase activity (B) over 48 h after spray treatment of 7-day-old wheat shoots with field rate concentration of cloquintocet mexyl (●), cloquintocet acid (○) and control (■).

**Fig. 5:**
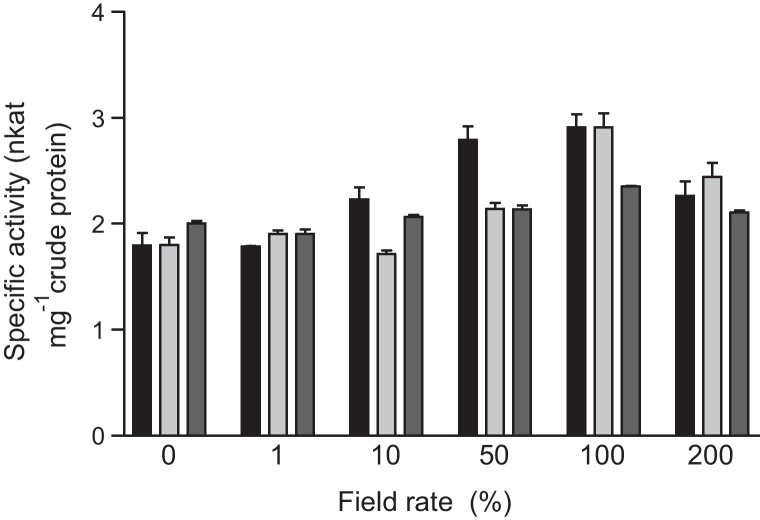
The response of GST activity towards CDNB in wheat shoots at 1 (■), 4 () and 7 () days after exposure to varying concentrations of cloquintocet mexyl. Values represent the means of duplicate determination with the standard deviation showing the extent of variation between replicates.

Based on the similarities in GST induction by the three safeners, cloquintocet mexyl was selected for more detailed study, not least because its herbicide protective effects could be uncoupled from the growth promoting activity observed with mefenpyr diethyl and fenchlorazole ethyl. To study the dynamics of GST induction in greater detail, untreated 7-day-old seedlings were sprayed with 10 mg L^−1^ cloquintocet mexyl at an application rate equivalent to that used for post-emergence application in the field. The induction of GST and GPOX activity was then determined over a 48 h period (Fig. 4). Both enzyme activities were significantly enhanced relative to controls within 8 h of treatment, with no further increase in specific activity determined over the remaining period up to 48 h. This showed that GST induction had occurred very quickly following exposure to the safener. To monitor uptake and metabolic fate during this period, wheat plants were treated with cloquintocet mexyl and extracts analysed by HPLC–MS. The results demonstrated that cloquintocet mexyl was rapidly hydrolysed to cloquintocet acid, which then accumulated during the induction period (data not shown). To determine if cloquintocet acid was active as a safener, the parent ester was hydolysed to yield the free acid which was then used to treat the wheat shoots, with the induction of GSTs and GPOX again monitored over a 48 h period (Fig. 5). The results obtained showed that the free acid gave essentially an identical fold-induction overall of both activities to the parent ester. However, unlike cloquintocet mexyl, the cloquintocet acid only gave maximal enhancement after 24 h of treatment. These results suggested that cloquintocet acid is in fact the active safener, with the ester effectively acting as an uncharged and hydrophobic precursor which assists in the rapid delivery of the compound across the waxy cuticle of the wheat leaf. As such the effective bioactivation of the safener is analogous to the uptake, hydrolysis and activation of herbicide esters (Cummins and Edwards, 2004).

A series of simple experiments were then performed to determine the dose responsiveness of safening using a single application of cloquintocet mexyl to 7-day-old seedlings. Normal field rate applications gave a 58% increase in GST activity 24 h after treatment, with a very similar response seen with the 50% field rate treatment. Increasing the safener dosage above field rate gave a smaller induction of GST activity. In each case, maximal induction was seen at 24 h, declining thereafter most noticeably at the lower application rates.

It was then of interest to study the GST induction response in different plant parts. Wheat seedlings were divided into leaves and meristematic tissues, with the leaves sub-divided into tips and middle sections. After normalising protein content, the samples were then analysed for CDNB-conjugating activity and phi, tau and lambda GST polypeptide content (Table 3). These results demonstrated that GST activity in the control plants was four-fold higher in the meristems than in the foliage, there being no differences between the tips and mid sections of the leaves. In terms of enzyme induction, the leaves were more responsive to safener than the meristems. Based on the Western blotting studies, whilst the GSTUs were present constitutively in both tissue types, the GSTFs were largely restricted to the meristems in the control plants. On safener treatment GSTFs began to accumulate in the leaves, whereas the GSTLs were only determined in the meristems (Table 3).

### 3.3 Protective effect of safeners in wheat towards soil-born pollutants

Whilst the protective properties of safeners towards herbicides are well described in cereals (Davies and Caseley, 1999), the potential for these compounds to enhance tolerance towards less selective phytotoxic chemicals has not been reported. In particular, the use of safeners to enhance chemical tolerance in plants grown in contaminated environments could be a very useful new tool in phytoremediation. To examine the potential utility of safeners in remediation applications, soil samples from a ‘real’ contaminated site located at a major oil refinery were used. In addition to oil residues, this soil also contained elevated levels of heavy metals (Table 1) and was taken from a site which had proven difficult to establish plants on in the field. In view of the differing activities on plant growth observed with traditional wheat safeners (Fig. 1, Table 2), the range of chemistries tested was expanded to include fenclorim which is used in rice and benoxacor and dichlormid which are used in maize (Davies and Caseley, 1999). These maize and rice safeners compounds had also been shown to elevate GST content in wheat (Edwards et al., 2005), making them suitable for comparative treatments with cloquintocet mexyl. Wheat seeds were soaked for 24 h in the four different safeners and then sown on the surface of the contaminated soil, along with non-polluted controls and germination and growth determined over a 14-day period (Table 4). In the absence of safener pre-treatment, none of the seeds germinated on the contaminated soil, whereas cloquintocet mexyl, benoxacor and dichlormid all gave similar results to those seen in the control soils. However, following germination the resulting shoot growth of the safened seedlings was greatly reduced compared with controls and by the end of the 14-day period the plants were showing signs of chlorosis. By 28 days all the seedlings exposed to the polluted soil were dead irrespective of treatment.

## 4 Discussion

Our results offer several new insights into the action of safeners in wheat. Using the induction of GSTs as a biomarker of detoxification we have demonstrated that distinct safener chemistries classes essentially induce identical responses in wheat seedlings, and that this effect is dose-dependent with respect to saturability and transience. Intriguingly, the two chemically related safeners, mefenpyr diethyl and fenchlorazole ethyl also provoked a growth enhancing effect. At the level of tissue selectivity, lambda GSTLs are induced only in the meristems, whilst the phi GSTFs accumulate more dramatically in the leaves. Finally we have shown that safeners can give a short lived protective effect to wheat during the early stages of germination and growth on soils contaminated with heavy metals and oil residues.

Based on the observed speed of the safener response, it is clear that these compounds must be rapidly recognised by an as yet unknown receptor system which initiates major changes in transcription (Riechers et al., 2010). It is therefore intriguing that distinct chemical classes of safeners give such similar detoxification responses, suggesting they are recognised by a common receptor system. Recent studies in Arabidopsis with a chemical series based on fenclorim were strongly suggestive of a selective protein-based receptor system, with minor changes in the reactivity and size of the safener strongly affecting GST-inducing activity (Skipsey et al., 2011). Similarly, a protein which selectively bound the safener dichlormid has been identified in maize, though its function remains unknown (Scott-Craig et al., 1998). Both fenclorim and dichlormid show the characteristics of protein alkylating agents, suggesting they need to covalently bind to the receptor target. In the case of the three safeners used in this study, there is no evidence that the parent compounds would serve as alkylating agents. Instead it would appear more likely that if they functioned via covalently modifying a receptor protein that this would occur following the formation of a reactive metabolite. In the studies with cloquintocet mexyl, we were unable to identify any downstream metabolites other than the free acid cloquintocet. By demonstrating that cloquintocet was as active at inducing GSTs as the parent ester, these studies did demonstrate that the acid, rather than the ester, was more likely to be the direct source of safener activity. However, the potential for a further and more chemically reactive downstream metabolite to be the active signalling agent is yet to be determined. The dose-dependence studies did show that there is a saturable threshold for the response, with higher concentrations only promoting a longer, rather than a larger, induction of GST activity. This is suggestive of a dynamic detection system whereby a minimal concentration of the active safening agent is required to sustain the response.

The differences between the distribution of members of the different GST families in leaves and meristems before and after safening were also intriguing and potentially important, with respect to the protective activity of these compounds. Safeners tend to protect cereals from herbicides such as the AOPPs which will predominantly act to inhibit fatty acid biosynthesis in the rapidly dividing cells in the meristems. Similarly, other ‘safened’ classes of herbicide such as the chloroacetanilides (fatty acid elongation inhibitors) and sulfonylureas (branched chain amino acid synthesis) will be most potent when acting on rapidly dividing meristematic cells. Studies in the related *T. tauschii* have suggested that the induction of tau GSTUs in the cells around the coleoptiles is critical in defining the protective activities of safeners (Riechers et al., 2003). It is therefore interesting that the induction of lambda GSTLs is also specific to the meristems. The GSTLs have a well established but as yet undefined relationship with safening in wheat (Theodoulou et al., 2003). Recently, we have shown that whilst GSTLs do not directly detoxify xenobiotics, they do have an important antioxidant function in which they can couple protective glutathione thiol and polyphenol metabolism (Dixon and Edwards, 2010). The current work lends further evidence for the functional importance of GSTLs in tolerance to chemically imposed stress in plants and suggests that such protection is cell type specific.

The finding that safeners can stimulate plant growth and provide some protection to wheat germinating in essentially toxic soil is also of potential interest. Several agrochemicals, most notably the strobilurin fungicides, are known to give growth promoting effects in wheat (Wu and von Tiedemann, 2001). Based on the studies presented here we would not anticipate the safeners to have any long term yield promoting effects, as the responses they invoke are too transient. Intriguingly, the growth promoting activity must be distinct from the suite of protective responses safeners induce in wheat as shoot growth was only stimulated by mefenpyr ethyl and fenchlorazole ethyl and not by cloquintocet mexyl. However, the production of thiols and antioxidant secondary metabolites associated with safening might help explain the protection these compounds exerted in the wheat plants grown in the contaminated soil. For example the glutathione known to accumulate on safening would be useful in counteracting metal ion toxicity, whilst the detoxification enzymes would provide some protection against organic pollutants. The results to date suggest that such protection only extends during the period in which the plantlets are growing from their own seed-born reserves. Nevertheless these observations with respect to growth promotion and enhanced chemical tolerance do point to new applications for safeners in agriculture and potentially phytoremediation.

## Figures and Tables

**Table 1:** Pollutant composition of test soil sample. Soil samples were provided from an oil refinery site which had been monitored for chemical content over a 5-year period using sequential extraction procedures (Zimmerman and Weindorf, 2010). Zinc equivalents refer to the contents of a range of metals used in alloy manufacture. Values quoted are averaged over a 5-year period for the site. Soil pH was determined as pH 5.8, with a water content of 24.2% (w/w).

Chemical	Availability (mg kg^−1^)
Boron	12.8
Copper	10.8
Nickel	24.8
Zinc	197.6
Zinc equivalents	417.3
Oil	3.1

**Table 2:** Effect of safeners on wheat shoot elongation and dry weight assimilation. Wheat seeds were imbibed for in 0.1% (v/v) acetone (control), or 10 mg L^−1^ solution of the safeners cloquintocet mexyl, fenchlorazole ethyl, and mefenpyr diethyl, followed by daily spraying with the same treatments after planting. Wheat shoots (50 per sample) were then measured for length and % dry weight determined at 7, 10, 12 and 14 days after sowing. Values shown are means ± standard deviations marked with * results being significantly different from the respective controls at a 95% confidence interval.

A	Days after sowing			
	7	10	12	14
	Mean height of shoots (mm)			
Control	96 (±23)	156 (±13)	195 (±10)	235 (±6)
Cloquintocet mexyl	97 (±14)	159 (±15)	195 (±8)	264 (±9)*
Fenchlorazole ethyl	106 (±10)*	181 (±15)*	230 (±11)*	264 (±15)*
Mefenpyr diethyl	101 (±10)	173 (±14)*	197 (±9)	245 (±10)*

B	Days after sowing			
	7	10	12	14
	Mean dry weight (mg) per 1 g fresh weight			
Control	90 (±5)	100 (±2)	100 (±2)	110 (±2)
Cloquintocet mexyl	90 (±4)	100 (±5)	110 (±3)	110 (±4)
Fenchlorazole ethyl	90 (±3)	110 (±3)	120 (±3)*	130 (±3)*
Mefenpyr diethyl	90 (±6)	110 (±2)	110 (±3)	120 (±3)*

**Table 3:** Induction of GSTs in different wheat plant parts based on (A) enzyme activity determination and (B) polypeptide composition, results and means ± standard deviations (*n* = 6) with * results being significantly different from the respective controls at a 95% confidence interval.

A	GST activity towards CDNB (nkat mg^−1^)	
	Control	Cloquintocet mexyl
Tip	0.46 (±0.01)	0.99 (±0.02)*
Middle	0.55 (±0.02)	1.01 (±0.01)*
Meristem	1.98 (±0.01)	3.05 (±0.02)*

B


**Table 4:** Effect of safeners on the germination and growth of wheat on control and pollutant contaminated soil.

Treatment	Pollutant	Germination rate (%)	Shoot growth (mm)
Control	−	70 ± 5	301 ± 36
	+	0	0

Benoxacor	−	55 ± 10	336 ± 27
	+	60 ± 10	49 ± 20

Cloquintocet mexyl	−	60 ± 0	377 ± 39
	+	60 ± 10	57 ± 10

Dichlormid	−	75 ± 5	340 ± 52
	+	60 ± 10	37 ± 10

Fenclorim	−	65 ± 5	353 ± 31
	+	45 ± 5	49 ± 5
